# Knowledge gaps and management recommendations for future paths of sustainable seaweed farming in the Western Indian Ocean

**DOI:** 10.1007/s13280-020-01319-7

**Published:** 2020-01-29

**Authors:** Maria Eggertsen, Christina Halling

**Affiliations:** Department of Ecology, Environment and Plant Sciences, Stockholm University, 106 91 Stockholm, Sweden

**Keywords:** Aquaculture, Coastal management, *Eucheuma*, Introduced species, *Kappaphycus*, Seaweed farming

## Abstract

Farming of eucheumatoid seaweeds is a widespread, promising activity and an important livelihood option in many tropical coastal areas as for example in East Africa, Western Indian Ocean (WIO). Compared to other types of aquaculture, seaweed farming has generally low impact on the environment. Nonetheless, there are potential direct or indirect negative effects of seaweed farming, such as introduction of alien species and changes in local environmental conditions. Although farming has been practiced in this region during several decades, the knowledge concerning the actual environmental impacts from faming non-native eucheumatoid haplotypes and consequently how to manage farming activities to mitigate those is highly limited. In this review, we provide a summary of the current scientific knowledge of potential direct and indirect negative environmental effects linked to eucheumatoid seaweed farming such as alterations of benthic macrophyte habitats and loss of native biodiversity. Furthermore, we highlight knowledge gaps that are of importance to address in the near future, e.g., large-scale ecosystem effects and farms as potential vectors of pathogens. We also provide a number of feasible management recommendations to be implemented for a continued development of environmentally sustainable seaweed farming practices in the WIO region, which includes spatial planning of farms to avoid sensitive areas and farming of native haplotypes of eucheumatoids instead of introduced specimens.

## Introduction

The growing demand for marine foods, commodities and products to sustain the world’s growing populations is one of the major drivers of increased exploitation of coastal zones worldwide. To meet these demands, aquaculture has been a steadily and rapidly growing industry since the end of the twentieth century (Subasinghe et al. 2009; Merino et al. 2012; FAO 2018). Within aquaculture, farming seaweed for consumption and food additives (Bixler and Porse 2011; Mouritsen et al. 2013; Tiwari and Troy 2015) animal feed or other applications (Zemke-White and Ohno 1999; Wijesinghe and Jeon 2012; Sangha et al. 2014) is among the fastest growing sectors (FAO 2018). During the last decades, the seaweed industry has more than doubled its production and evolved into a multi-billion dollar industry with an annually production of approximately 30 billion tons (Mac Monagail et al. 2017; FAO 2018).

A significant part of the global seaweed production constitute of *Eucheuma denticulatum* and *Kappaphycus alvarezii*, two species of tropical red algae, farmed for their content of carrageenan (Renn 1997; Bixler and Porse 2011; Buschmann et al. 2017; FAO 2018). Both species possess a relatively high growth rate (4–12% day^−1^) and can be farmed with simple and inexpensive methods (Bryceson 2002). Farming of eucheumatoid seaweeds was initiated in 1969 in the Philippines, South East Asia (SEA), and later introduced in Indonesia (Valderrama et al. 2013). These two countries are still the leading producers on the global market, although farming practices have spread to other tropical countries such as Malaysia, India, and the Solomon Islands (Valderrama et al. 2013; Phang et al. 2019).

In East Africa (EA), commercial harvesting of native wild eucheumatoid seaweeds was practiced in Tanzania during several decades (Mshigeni 1984). Due to steep declines in natural seaweed populations in the late 1980s, either because of overexploitation, natural fluctuations, or a combination of both (Mshigeni 1984), farming of SEA eucheumatoids was successfully established in Zanzibar 1989 and from there spread to other parts of the Western Indian Ocean (WIO) region (e.g., Kenya and Mozambique) (Msuya et al. 2014). The high growth rate of SEA eucheumatoids was the primary reason why non-indigenous stock of *Eucheuma denticulatum* and *Kappaphycus alvarezii* were introduced for farming purposes, although both species are native to and were already present in Tanzanian waters (Lirasan and Twide 1993; Tano et al. 2015).

In many low-income countries, initiation of seaweed farming has been considered as a management strategy, introducing an alternative livelihood option among resource poor coastal communities to decrease fishing pressure (Sievanen et al. 2005), empower women, and reduce poverty (Msuya 2006; Valderrama 2012; Mantri et al. 2017). Also, the rather low-technique farming methods and low investment costs are added to its attraction. For example, the most commonly used method in East Africa is the ‘off-bottom method’ using fronds of algae tied to mono-filamentous lines between wooden sticks pegged into the bottom in the intertidal zone (Ask and Azanza 2002; Msuya et al. 2014). Seeding material is simply taken as vegetative cuttings from the last harvest (Luxton 1993).

**Fig. 1:**
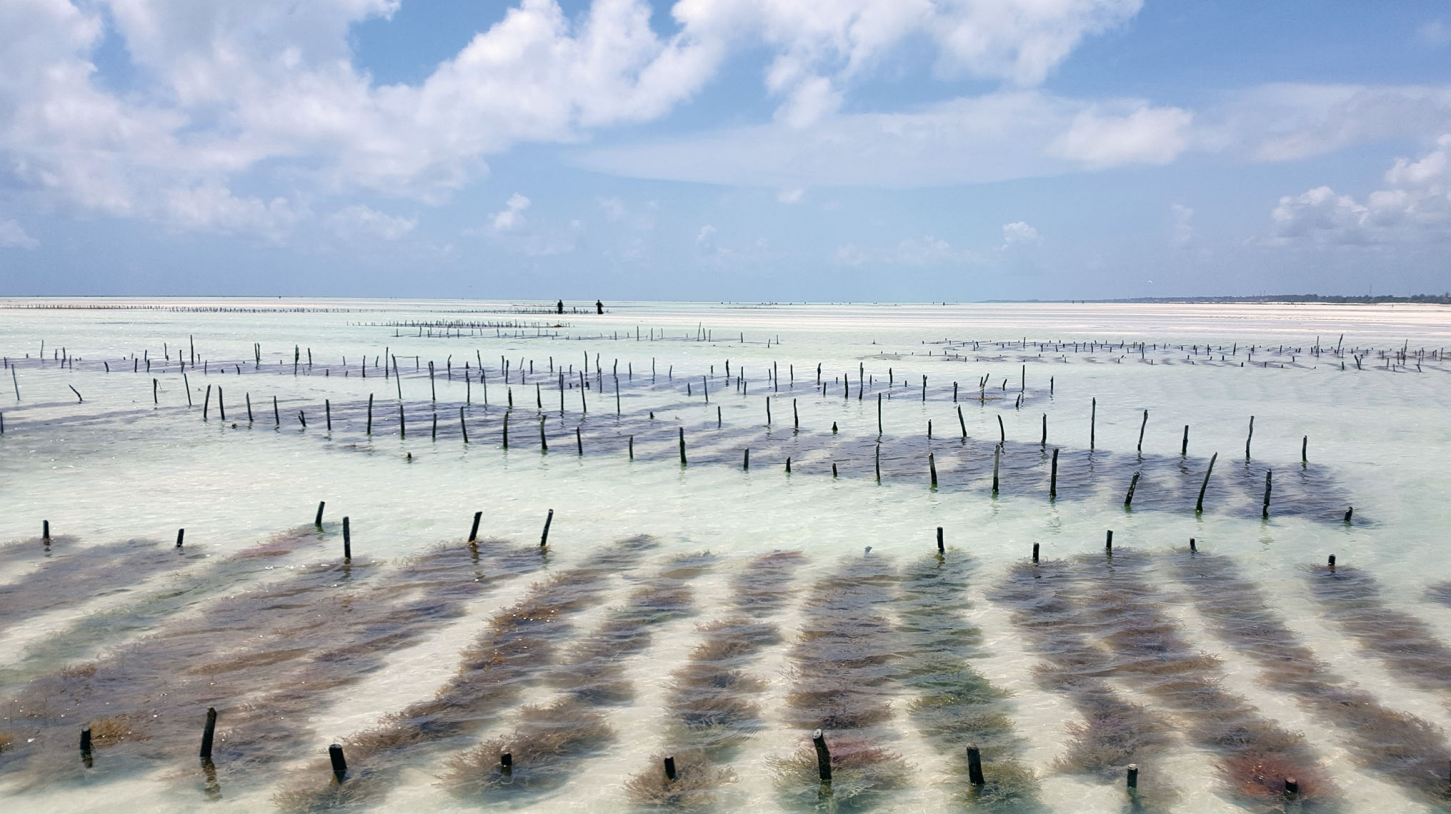
Seaweed farms of *E. denticulatum* in Paje, east coast of Zanzibar. Photo courtesy of C. Halling

There is evidence that eucheumatoid seaweed farming has improved socio-economic status of coastal communities where other livelihood options are scarce, for example, in cases where marine resources are reduced or depleted due to overexploitation (Rönnbäck et al. 2002; Msuya 2006; Valderrama 2012). However, studies performed in SEA have shown that seaweed farms seldom remove fishing pressure but rather function as a supplementary income (Sievanen et al. 2005; Hill et al. 2012). Currently, Tanzania (including Zanzibar) is a significant but relatively small producer of mainly *E. denticulatum* (Fig. 1) as *K. alvarezii* has been more susceptible to diseases and epiphyte infestations (Msuya et al. 2014). In 2015, Tanzania (mainly Zanzibar) exported 14 000 dry tons, compared to the largest producer Indonesia which exported 110 000 tons the same year (Porse and Rudolph 2017; FAO 2018). Decreased production due to epiphytes and pathogens in combination with declining market prices has, during the last decade, led to reductions and abandonments of farms (Msuya et al. 2014). The low profitability is also a result of the lack of local processing. Although farmed in tropical regions, the dried raw material is usually transported to Europe or USA for refining and extraction, and it is also here the largest monetary value increase is occurring (Bryceson 2002; Valderrama et al. 2013).

This management and decrease in farming activities is unfortunate for the WIO region as it impedes the regional seaweed assessment and market development, despite the continuous massive increase in global and future demand for seaweed and seaweed products (Buschmann et al. 2017). Such demand implicates a great economical potential for the countries in the WIO and other coastal countries in the tropics within the seaweed aquaculture sector if taken into account (FAO 2018). The Malaysian seaweed industry development is a good example of how rather limited but targeted governmental initiatives and investments can substantially increase benefits. By strengthening the seaweed industry by for instance establishing seaweed farming directed specification standards and code of practices as well as providing training and some improved technical and infrastructure support, the seaweed industry is currently in priority and expanding (Phang et al. 2019). Such a targeted strategy for seaweed management could be of great importance within the WIO as seaweed farming is, despite the current drawbacks, an important income source for thousands of households in coastal Tanzania (F. Msuya pers. comm.; Charisiadou et al. in prep).

Compared to other types of aquaculture, such as fish and shrimp farming, seaweed farming has a significantly lower impact on the marine environment. Concerning farming of *E. denticulatum* and *K. alvarezii,* no freshwater, fertilizers or antibiotics are used, and there are no detrimental nutrient effluents (Halling et al. 2013). However, as farms are usually placed in shallow water areas in the intertidal, they may overlap and affect, directly or indirectly, important habitats such as seagrass beds and coral reefs (Eklöf et al. 2006a; Hedberg et al. 2018). Also, spread of non-indigenous species from farms might be a potential problem. Because these seaweeds are farmed in open systems, there is always the risk of breakage and spread of farmed seaweed fronds into surrounding habitats. Introduction of foreign species that might become invasive has been described as a major threat to marine biodiversity (Bax et al. 2003; Courchamp et al. 2017), and seaweed aquaculture is considered a major vector of unintentional introductions worldwide (Schaffelke and Hewitt 2007).

Introductions of SEA haplotypes of *E. denticulatum* and *K. alvarezii* outside farms have been reported from multiple countries (Rodgers and Cox 1999; Barrios et al. 2007; Ferreira et al. 2009; Halling et al. 2013; Sellers et al. 2015; Cabrera et al. 2019), and in some cases, they have become invasive, mainly resulting in detrimental effects on reef-building corals, due to smothering and shading, eventually causing extensive coral die-offs (Conklin and Smith 2005; Barrios et al. 2007; Chandrasekaran et al. 2008; Bindu and Levine 2011). In certain locations in India, cultivation of *K. alvarezii* was temporarily banned (Kamalakannan et al. 2010) and both India and Hawai’i have had state funded initiatives removing ‘escaped’ seaweeds from coral reefs (Kamalakannan et al. 2014; Neilson et al. 2018). In East Africa, molecular studies have confirmed that the farmed introduced eucheumatoids are genetically distinct from the native ones (Zuccarello et al. 2006; Halling et al. 2013).

A certain aspect of the spread and introductions of farmed seaweeds is the risk of unintentional selection on certain traits resulting in domestication of crops and consequently potential modifications of physical responses (Zohary 2004; Guillemin et al. 2008; Valero et al. 2017). This has been observed for the red algae *Agarophyton chilensis* in Chile, where farmed individuals were significantly more tolerant to temperature variations compared to natural/’wild’ populations of the same species (Usandizaga et al. 2019). Such domestication of farmed populations, caused by the repetitively vegetative propagation of cuttings for farming, favors traits for high growth also under wide and stressful conditions, making them resilient to environmental changes, thus at the cost of genetic diversity (Guillemin et al. 2008), but not necessarily in productivity (Usandizaga et al. 2018; 2019). This kind of wide-ranging resilience is often characteristic also for species/haplotypes being invasive (Richards et al. 2006).

In general, there are limited data on environmental effects caused by tropical seaweed farming (direct or indirect by, for example, the introduction of non-indigenous species/haplotypes). The aims of this review are therefore to *i)* summarize the current scientific knowledge of direct and indirect environmental impacts of eucheumatoid farming and *ii)* identify important knowledge gaps concerning ecological effects, by using Zanzibar in Tanzania as case study, with special focus on the potential effects, concerning introductions of foreign strains. The identified knowledge gaps are used for recommending management or future research priorities aiming for mitigation of negative effects of seaweed farming both generally and specifically for the WIO region.

We define direct effects as the impacts of seaweed farming on the surrounding habitat caused either by the physical structure of the farms (such as shading of benthos or destruction of habitat due to the construction of farms) or by farming activities (such as trampling when maintaining farms or harvesting). Indirect effects are identified as environmental impacts originating from seaweed farms but not necessarily in direct connection with farms, such as introduction of non-indigenous haplotypes and establishment on coral reefs by seaweeds escaped from farms.

## Environmental impacts of farming and introduction of sea eucheumatoids in the WIO—direct effects

### Impacts of seaweed farming on seagrass assemblages

Because Zanzibari seaweed farms are often placed on seagrass meadows (Hedberg et al. 2018), efforts have been made to evaluate the effects of seaweed farming on the seagrass assemblages in the area. Seagrasses are highlighted as key habitats for many fish and invertebrates by providing both nursery and adult habitats and also by constituting important fishing grounds for local communities (Gullström et al. 2002; de la Torre-Castro and Rönnbäck 2004; Dorenbosch et al. 2005; Nordlund et al. 2018). Loss of seagrass areas could therefore have major negative consequences, not only for the marine fauna, but also upon the people that are directly or indirectly dependent on the ecological goods and services they generate (Cullen-Unsworth et al. 2014; Nordlund et al. 2018).

All studies evaluating seaweed farming on seagrass communities have reached similar results; directly beneath seaweed farms compared to control areas, seagrasses were sparser and leaf biomass were lower (Eklöf et al. 2006a; Lyimo et al. 2006), or seagrasses had disappeared completely (Mallea et al. 2014) or macrofauna was reduced and less diverse (Ólafsson et al. 1995; Eklöf et al. 2005). This is probably due to shading and/or competition for nutrients and CO_2_ (Eklöf et al. 2005). Shading has also been shown to decrease carbon sequestration capacity of seagrasses (Dahl et al. 2016). Because farmers are constantly working in the cultivation areas, decreases in seagrass biomass or less shoot densities can also originate from mechanical damage (trampling, deployment of poles in bottom substrate) and direct removal of seagrass shoots by the seaweed farmers (Lyimo et al. 2006). However, these studies were made on farm-patch scale, i.e., directly beneath farms (except for Lyimo and colleagues who also measured seagrass cover in corridors between farms), so there are currently no data evaluating if and how seaweed farms and farming affect seagrass ecosystems on a larger scale (for example, in a whole bay system) and in a longterm perspective (time of recovery etc.). Hence, with the current knowledge, predicted effects of seaweed farming on seagrass assemblages can only be dependent on the total farmed area (patch level).

### Impacts of seaweed farming on fish and fisheries

In parts of the world where eucheumatoid seaweed farming is extensive and large scale (e.g., the Philippines, Malaysia, Indonesia), a positive relationship between siganid fish catch and harvested volume of seaweeds is found (Hehre and Meeuwig 2016). Fish from the Siganidae family, which is an important food fish in East Africa (de la Torre-Castro and Rönnbäck 2004), occur frequently in seaweed farms (Bergman et al. 2001), and also feed on *E. denticulatum* (Eggertsen et al. 2019), suggesting that seaweed farming in Zanzibar may similarly benefit siganid fisheries. However, this has not been observed in small-scale production nations such as Tanzania (Hehre and Meeuwig 2016), but since this study was based on commercial fish catches only (meaning that artisanal and recreational fishing is excluded), local effects on fish assemblages might not have been possible to detect.

In Kenya, seaweed farms have been shown to attract herbivorous fish, which are feeding on fronds (Anyango et al. 2017), and anecdotal data from Mafia Island (Tanzania) tell that invertivorous fish from the Lethrinidae family migrate to seaweed farms where they feed on seaweed-associated epifauna. However, the impact of seaweed farms on fish abundance is ambiguous, as a study from The Philippines concluded that seaweed farms also might have a negative effect on fish assemblages (Hehre and Meeuwig 2015), so more research is needed to understand the mechanisms behind these patterns.

A positive response in siganid fish catches was observed by Eklöf et al. (2006b) in Zanzibar as fish traps in seaweed farms had higher catches than fish traps placed on sand, and similar catch in numbers as fish traps placed in seagrass. Species identity was slightly different between the two vegetated habitats, siganids dominated the seaweed farm traps while a labrid (*Cheilinus chlorurus*) was more common in catches from seagrass areas (Eklöf et al. 2006b). Furthermore, in another study from Zanzibar, seaweed farms were shown to host higher fish abundances and higher species richness than control areas in one location, but not in another (Bergman et al. 2001). This is probably due to site-specific differences, where the latter site had a higher structural complexity than the former, so that seaweed farms added a significantly higher structural complexity to the low-complexity site. Structural complexity is a well-recognized factor in influencing near-shore fish assemblages by providing habitats rich in food and shelter (Bell and Galzin 1984; Nagelkerken and Van der Velde 2002; Almany 2004).

Hedberg et al. (2018) expressed concern that seaweed farms might compete with and disturb small-scale fisheries since these activities sometimes overlap spatially. However, no such conflict was heard of when asking seaweed farmers in Zanzibar (I. Bryceson, pers. comm.), and fishers at Mafia Island said that they sometimes deliberately put fish traps and also fished with line inside farms as they are considered good fishing grounds (pers. obs.). Therefore, at some locations in the WIO, there might be a synergistic relationship between small-scale fisheries and seaweed farms (similar to patterns observed in Asia by Hehre and Meeuwig 2016), but this has to be further evaluated.

### Additional environmental impacts of seaweed farming

There are little data available on environmental impacts beyond what is listed above of eucheumatoid seaweed farming on surrounding and adjacent ecosystems. Large-scale seaweed farms could potentially disturb feeding activities of dugongs, and possibly entangle them (Poonian and Lopez 2016), but as dugongs are extremely rare in Tanzanian waters and probably absent from Zanzibar where the most intense seaweed farming is occurring (Muir et al. 2003), this is an unlikely consequence of seaweed farming in the WIO.

Increased oxygen levels around seaweed farms due to photosynthesis and nutrient removal are other effects which likely occur, but literature is limited on this topic. Eucheumatoids have been shown to be efficient in nitrogen uptake from the surrounding seawater (Dy and Yap 2001), which might be both negative or positive: negative through competition with other organisms and positive by performing ecosystem services such as removing nutrients from the water column and thus increasing water quality and mitigate eutrophication (Xiao et al. 2017). In experiments with integrated aquaculture systems, both *E. denticulatum* and *K. alvarezii* have been shown to be efficient in nutrient uptake, indicating a possible bioremediation potential for these species (Mwandya et al. 2001; Rodrigueza and Montaño 2007; Bindu and Levine 2011). A positive effect as such may be considerable when farming large volumes; for example, large-scale farming of *Gracilaria lemaneiformis* resulted in an inhibiting effect on the bloom of certain harmful algae (Yang et al. 2015). On the other hand, this may indicate a risk that large-scale seaweed farming under certain circumstances can cause nutrient depletion and induce competition with native communities (Préat et al. 2018). Such depletion may also negatively affect the productivity of the farmed species itself. However, in open water conditions and proper management with sufficient water circulation, this should not be the case.

Introduced seaweeds and/or haplotypes could be a vector of pathogens, parasites, or other non-native species (Cottier-Cook et al. 2016; Buschmann et al. 2017). Likewise, large-scale monocultures can facilitate the growth of pathogens, which potentially could be transferred to wild algal populations (Cottier-Cook et al. 2016).

## Indirect effects

### Impacts of seaweed farming on native seaweed populations

Introduced non-native seaweeds are associated with the risk of becoming invasive in their new habitat, changing environmental conditions and even outcompeting native flora and fauna (Bax et al. 2003; Schaffelke and Hewitt 2007). Other potential consequences such as alterations of ecosystem functions can occur on different levels within the recipient system, for example, changes in community composition (Davidson et al. 2015), productivity (Sagerman et al. 2014), habitat complexity (Veiga et al. 2014), and biodiversity (Casas et al. 2004; Schaffelke and Hewitt 2007).

Reductions in species richness of native seaweed communities have been reported as a consequence of introductions of non-native seaweeds (Schaffelke and Hewitt 2007). Similar studies have not been performed in the WIO area, and studies on impacts on recipient macrophyte systems have mainly been concerned with seagrasses (see Eklöf et al. 2005, 2006a; Lyimo et al. 2006), although there is evidence that seaweed farming can reduce native seaweed biomass in seagrass habitats (Eklöf et al. 2006a). In general, there is little scientific information regarding natural seaweed habitats and ecological functions from this geographical area (but see Tano et al. 2016, 2017).

Because both *Eucheuma denticulatum* and *Kappaphycus alvarezii* are indigenous to the WIO area, detection of a possible invasion of the farmed SEA haplotypes (we will hereafter in this review refer to these as ‘haplotypes’ of SEA or EA types) is hampered by the difficulty to visually tell the different strains apart. However, both Halling et al. (2013) and Tano et al. (2015) could show by using molecular methods that SEA haplotypes have spread from farms and are now to be found in Zanzibari macroalgal/reef habitats. If the introduced eucheumatoids are competing with native populations is currently unknown, but growth rate experiments have shown that SEA *E. denticulatum* generally possess a higher growth rate than native *E. denticulatum* (Halling, unpubl.), indicating that this could be a potential risk. Similarly, introduced eucheumatoids could also potentially compete with other seaweed species, for example, important habitat-forming species such as *Sargassum* spp.

Two introduced SEA haplotypes of *E. denticulatum* are identified in Tanzanian waters; E13 which is currently farmed, and E32 which has only been found in the wild but probably has been introduced previously through farming (Halling et al. 2013; Tano et al. 2015) and at least 7 EA haplotypes (Zuccarello et al. 2006; Halling et al. 2013; Tano et al. 2015; Eggertsen et al. unpubl.). So far, fewer haplotypes of *K. alvarezii* have been detected in Tanzanian waters: two native and two introduced. The native ones have been collected both in the wild and in the farms and the introduced virtually only in farms (but see Halling et al. 2013). There is recent information that farming of a third eucheumatoid species, *K. striatum,* has been initiated in Zanzibar as an alternative to *K. alvarezii* (F. Msuya pers. comm.). This farming is yet highly limited and the geographical origin of the cultivars is not known. In Zanzibar (Unguja Island), introduced SEA *E. denticulatum* strains currently dominate eucheumatoid populations around the island, also in areas with very little seaweed farming (Tano et al. 2015). However, there is no doubt that the SEA haplotypes of *E. denticulatum* in Tanzania originates from the seaweed farming (Halling et al. 2013; Tano et al. 2015).

No sexually reproducing SEA individuals have yet been found in Tanzania (Tano et al. 2015) which also explains, proven by its absence, why hybridization between EA and SEA haplotypes might not be an issue. The absence of SEA *K. alvarezii* in the wild could partly be explained by its higher susceptibility to epiphyte infestations and die-offs, resulting in much smaller cultivation volumes (Hayashi et al. 2010) and consequently fewer escapees.

The higher growth rate compared to EA haplotypes and the absence of sexually reproducing individuals among the SEA haplotypes found in Tanzania may indicate a possible domestication of the farmed SEA haplotype (E13). This is a process which has been observed for other farmed seaweeds, e.g., *Agarophyton chilensis* in Chile (Guillemin et al. 2008; Usandizaga et al; 2018; 2019), but not yet for eucheumatoids.

### Impacts of seaweed farming on reef-building corals

An issue highlighted as one of the most problematic consequences of non-native eucheumatoid introductions is competition with and smothering of scleractinian corals (Conklin and Smith 2005; Kamalakannan et al. 2010; Neilson et al. 2018). Currently, no such cases have been reported from the WIO area, but this is potentially a result of limited research resources and consequently no monitoring of reefs, rather than a non-existing phenomenon. In both Hawai’i and India, where detrimental effects on reef-building corals have been observed, introduced eucheumatoids have spread by fragmented pieces and successfully established on reefs (Conklin and Smith 2005; Chandrasekaran et al. 2008; Kamalakannan et al. 2010). One of the most well-studied cases of eucheumatoid introductions with severe indirect environmental consequences is the one in Kane’ohe Bay, Hawai’i, (see, e.g., Rodgers and Cox 1999; Conklin and Smith 2005; Conklin et al. 2009). The main effects were identified as direct competition, smothering and killing of reef-building corals (Bahr et al. 2015), illustrating that eucheumatoids have the ability to compete with other benthic organisms and introductions are not to be neglected.

Eucheumatoids are documented to have the ability to adhere to coral tissue, creating extremely strong attachments, and also to regrow from minute fragments (Conklin and Smith 2005; Kamalakannan et al. 2014) thus making them successful in colonizing new substrate. Kamalakannan et al. (2014) described the overgrowth of *Acropora* spp. colonies by *K. alvarezii* in the Gulf of Mannar, India, and coral recovery with and without algal overgrowth after a bleaching event, where infested reefs did not recover. By monopolizing settling substrate, seaweeds can inhibit coral recruitment, a phenomenon that has been reported for several seaweed species (Kuffner et al. 2006). As coral recovery is dependent on successful recruitment, introduced eucheumoid seaweeds might be able to cement a reduction in coral cover, especially after large-scale coral die-offs.

Scientific studies from Hawai’i and India are mainly concerning SEA *K. alvarezii*, but as field identification sometimes can be difficult, it cannot be excluded that SEA *E. denticulatum* is also involved. The SEA haplotypes of *E. denticulatum* found in Zanzibar (E13 and E32) are genetically similar or identical to the invasive haplotypes found in Hawai’i (Zuccarello et al. 2006), why there is a potential risk that a similar coral competition situation could occur also in the WIO.

## Major scientific knowledge gaps concerning potential environmental effects of WIO seaweed farming

### The question of scale and its importance for environmental impacts by seaweed farming

To correctly emphasize environmental effects of seaweed farming, it is of critical importance to consider both scale and intensity as well as identify economical and environmental threshold values for farmed areas, which are highly dependent of site-specific features. Currently, such information is lacking. As seaweed farming is dynamic in space and time (seasonal and between years), it is difficult to make precise estimations on how large areas of the coastal zone that are affected by seaweed farming. The actual farming area and its potential environmental impact is highly dependent on the economically viability and profitability of farming at a certain time, as well as farmers access to suitable farming areas. In a study from 2016, seaweed farming in Zanzibar occupied only 15% of total suitable farming areas, constituting ‘corridors’ along the coast in villages where farming was practiced (Hedberg et al 2018). Even if 15% of a distinct area within a seascape can be considered a major impact, the small-scale seaweed farming in the WIO should, however, be regarded as an activity with overall low environmental impact. One of the largest farming sites (Jambiani) amounts to 25.7 ha which is considerable small when compared to one of the largest farming sites in the Philippines, the Sitangkai, Tawi-Tawi that covers an area of 10 000 ha. There are surprisingly few studies on environmental effects from seaweed farming from the Philippines and Indonesia despite the higher intensity and production. This lack of studies and reports on environmental impact might be an implication on the non-existing environmental consideration concerning seaweed farming among management. Compared to many other coastal activities with relatively high negative impact on the environment, seaweed farming might seem reasonable.

### Knowledge gaps concerning competition of introduced eucheumatoids vs indigenous taxa—indirect effects

In one of the few studies of natural eucheumatoid populations pre-farming time in Tanzania, Mshigeni (1984) described *Eucheuma denticulatum* as the most common eucheumatoid algae in Tanzanian rocky shallow water habitats. However, because no inventory studies on cover or densities of native eucheumatoid populations were performed, it is impossible to draw any conclusions about whether current densities are abnormal, whether SEA haplotypes have swamped and replaced EA ones, or if they are simply coexisting. Studies of geographical origin/population structure of eucheumatoids in the wild have been performed on a limited stretch of the WIO coast (Halling et al. 2013; Tano et al. 2015), but introductions might occur all along the East African coast where seaweed farming is or has been practiced. This is an important knowledge gap to fill when investigating general impacts of farming of non-indigenous *E. denticulatum* which also links to the question on which spatial scales environmental effects might occur. Likewise, it is unknown if the introduced haplotypes are competing with other seaweeds for substrate, such as the habitat-forming *Sargassum* spp. and more research would be needed to determine this.

It is not established exactly how corals are affected by overgrowth of eucheumatoid seaweeds. Conklin and Smith (2005) described infested corals as slightly bleached when shaded and necrotic where algae attached, but if the coral was already dead prior to attachment or if this was inflicted by the algae was not specified. Chandrasekaran et al. (2008) concluded that invasive *Kappaphycus alvarezii* seems to prefer *Acropora* spp. as growth substrate over *Pocillopora* spp. but it is not clear if this depends on structural complexity only or species-specific coral–algae interactions. Several studies have shown that corals can be negatively affected by chemical compounds emitted by seaweeds (Shearer et al. 2012; Thurber et al. 2012; Bonaldo and Hay 2014), but whether this is also the case with eucheumatoid seaweeds is not known.

### Scientific knowledge gaps to address in the WIO area

A majority of the studies of environmental impacts inflicted by seaweed farming in the WIO area are focusing on direct effects of seaweed farming (Table 1). However, indirect effects such as interactions with native eucheumatoid populations are important to acknowledge. Evidence from Zanzibar indicates that there are established non-native populations of *E. denticulatum* in the wild originating from farming activities (Halling et al. 2013; Tano et al. 2015), and there is a need to evaluate if this is consistent over the region. Important knowledge gaps to address would also be how and if introduced *E. denticulatum* is competing with native eucheumatoid populations in the WIO area. Similarly, potential competition with other native algae such as *Sargassum* spp. should be investigated, including studies over different seasons. In addition, the possibility of domesticated traits within SEA haplotypes and how these may affect its ecological performance, such as coping with anthropogenic impacts (Usandizaga et al. 2019), should be examined from different perspectives (e.g., for continuous production and for the issue of being highly competitive).

**Table 1:** Potential environmental impacts of eucheumatoid seaweed farming and knowledge gaps in the WIO area

Environmental impacts	Category	Location	References	Studies in the WIO area
Seagrass biomass loss (decreases in shoot density, shoot length and leaf growth rate)	Direct	Zanzibar (Unguja Island)—Tanzania, Cuba	Eklöf et al. (2005, 2006a), Lyimo et al. (2006), Mallea et al. (2014)	3
Large-scale effects, e.g., on seagrass systems in a bay system where farming is occurring	Indirect	–	–	None
Macroalgal biomass loss beneath farms (in seagrass meadows)	Direct	Zanzibar (Unguja Island)—Tanzania	Eklöf et al. (2006a)	1
Effects on meiofauna/invertebrate macrofauna (changes in nematode assemblages, decreases in bivalve abundances)	Direct	Zanzibar (Unguja Island)—Tanzania	Ólafsson et al. (1995) and Eklöf et al. (2005)	2
Recovery rates of seagrass/invertebrate fauna (post-farming)	Direct	–	–	None
Temporal effects on seagrass/invertebrate communities beneath farms	Direct	–	–	None
Effects on fisheries (changes in species assemblages in catches and numbers)	Direct	Zanzibar (Unguja Island)—Tanzania, Kenya, Indonesia, Malaysia, Philippines, Fiji	Eklöf et al. (2006b), Hehre and Meeuwig (2016) and Anyango et al. (2017)	2
Effects on fish assemblages (changes in species assemblages, decreases in species richness and biomass)	Direct	Philippines	Hehre and Meeuwig (2015)	None
Effects on reef-building corals (by overgrowth and smothering by escaped seaweeds)	Indirect	India, Oahu—USA, Venezuela	Conklin and Smith (2005), Barrios et al. (2007), Chandrasekaran et al. (2008), Kamalakannan et al. (2010) and Neilson et al. (2018)	None
Effects on recruitment of benthic taxa (e.g., corals and seaweeds by monopolizing settling substrate)	Indirect	–	–	None
Effects on natural seaweed habitats (e.g., *Sargassums* spp. assemblages)	Indirect	–	–	None
Effects on indigenous eucheumatoid populations	Indirect	–	–	None
Seaweed farms as vectors of pathogens/epiphytes, spread of associated introduced species	Indirect	–	–	None

Long-term studies on impacts from farming on other ecosystems are lacking, i.e., if a seagrass system will recover after farming is terminated. Additional useful information would be how rapidly a newly established seaweed farm suppress the underlying biotic communities. The thorough studies by Ólafsson et al. (1995) and Eklöf et al. (2005, 2006a) on direct effects on seagrass communities and infauna caused by seaweed farms compare already farmed patches with non-farmed patches. Knowledge on what temporal scales these processes operate would be desirable, because depending on that time scale, rotation of farmed patches might be an option.

Because coral overgrowth by eucheumatoids has been problematic in other locations, there is a need to investigate if introduced *E. denticulatum* is competing with and/or overgrowing reef-building corals in the WIO area, similar to what has been observed in other geographical locations. Also, the issue of whether introduced seaweeds suppress coral recruitment by monopolizing settling substrate should be investigated. The WIO area has been subjected to some severe coral bleaching events (1998, 2010, 2016), resulting in large-scale coral mortality in shallow water areas (McClanahan et al. 2007; Eriksson et al. 2012; McClanahan 2017; Obura et al. 2017; Cowburn et al. 2018), thus opening up space for algal colonization. Recruitment is essential for the recovery of reefs, and as coral bleaching might be more frequently occurring due to climate change, factors limiting recruitment should be avoided.

To reduce the spread of SEA haplotypes of eucheumatoids in the WIO area, it is recommended that more efforts should be made to identify EA haplotypes of *E. denticulatum* (among other native eucheumatoids), which are suitable/profitable for farming purposes. In that way, there is a limited risk of loss of genetic diversity among indigenous *E. denticulatum* populations. Also, as there are currently no observations of detrimental effects of indigenous *E. denticulatum* on corals (but this has to be evaluated further), farming of EA seaweeds would be beneficial also from that perspective.

Last but not least, efforts should be made to try to identify extrinsic factors (positive and negative) influencing and regulating spread of SEA *E. denticulatum* from farms. Threshold values for dispersal distances and environmental variables that could affect this would be important information to build on, for example, within management efforts. More or less suitable farming locations could potentially be identified by using this type of data.

## Recommendations for management

Different management solutions have been used to reduce impacts by introduced eucheumatoids in different locations (Kamalakannan et al. 2014; Castelar et al. 2015; Neilson et al. 2018). In 2003, Ask et al. stated that no negative environmental effects had been observed due to introductions of eucheumatoids for farming purposes. Today, we know that environmental impacts caused by introduction as such do exist (Table 1).

Top-down control (outplants of sea urchins and/or mechanical removal) might be one of the most efficient management strategies in already infested areas and has been practiced in both Hawai’i and India with various success (Kamalakannan et al. 2014; Neilson et al. 2018). Herbivory by fish and invertebrates might also have the ability to reduce the biomass of eucheumatoids and potentially decrease the spread of introduced seaweeds. Top-down control on algal assemblages by herbivores is a process documented in multiple locations (see, e.g., Burkepile and Hay 2006; Vergés et al. 2009; Taylor and Schiel 2010), although not specifically tested for eucheumatoids. Bioremediation using sea urchins has been partly successful against invasive *E. denticulatum* in Hawai’i (Neilson et al. 2018), but this has not been tested in the WIO area and would need to be investigated further. One concern is the overgrazing and consequently loss of seagrass meadows caused by sea urchins, which is observed in several locations in the WIO (Eklöf et al. 2008). Sea urchin ‘outplantings’ might therefore not be feasible in this region.

In Brazil, where farming of *Kappaphycus alvarezii* has been tested in the southern part of the country, regular environmental monitoring was suggested by the state authorities, but invasions have not occurred, possibly due to low temperatures in the winter and lack of potential substrate (Castelar et al. 2009). A complete ban of farming has been recommended in the northeast of Brazil, where environmental conditions are more favorable for eucheumatoids (such as temperatures and presence of suitable habitats in the form of coral reefs) (Castelar et al. 2015) and in some locations in India (Kamalakannan et al. 2014).

A complete ban might not be the best option in areas where economically vulnerable people are depending on income generated by farming of introduced seaweed haplotypes, especially in locations that lack other livelihood alternatives as in the WIO region (Fröcklin et al. 2012). Also, if negative environmental effects are negligible, there is no reason to ban small-scale farming of SEA haplotypes. Benefits generated by seaweed farming have to be weighed against these possible negative environmental effects (also in comparison with other relevant livelihood alternatives), which may vary depending on location. Likewise, regular monitoring of spread in adjacent habitats is not very feasible as an option in countries with limited economic capacity. However, in the following section, adaptive management actions are suggested that might prevent spread of introduced seaweeds and direct environmental effects of farms and mitigating the potential negative effects from seaweed farming.

### Management recommendations in the WIO region

Direct and indirect effects of seaweed farming in the WIO might need to be managed slightly different (Table 2). For direct effects, methods such as spatial planning, size restrictions, farming plot placements, and rotation of farming plots might be feasible whereas indirect effects might need more site-specific recommendations and more research performed. Based on the present review, indirect effects are also the least studied in the WIO area. To mitigate potential negative environmental effects of eucheumatoid cultivation in the WIO region, the following measures based on information derived from the current literature are suggested.

**Table 2:** General management suggestions to mitigate potential negative direct and indirect effects on the environment by seaweed farming

Environmental impacts	Category	Management suggestion
Negative effects on seagrass communities	Direct/indirect	Avoid placing seaweed farms on seagrass meadows (especially meadows consisting of more sensitive seagrass species such as *E. acoroides*) to mitigate trampling, mechanical damage, and shading. Avoid covering all seagrass meadows in an area with seaweed farms. Rotation of farming patches might be an option
Negative effects on meiofauna/invertebrate macrofauna	Direct	Avoid covering too extensive areas with seaweed farms. Rotation of farming patches might be an option
Negative effects on reef-building corals (shading, smothering)	Indirect	Avoid placing seaweed farms in the vicinity of coral reefs (identification of threshold values necessary) with a high degree of structural complexity (e.g., branching corals). Farming of EA *E. denticulatum* only is recommended
Negative effects on recruitment of benthic taxa	Indirect	Avoid placing seaweed farms in the vicinity of areas with a high degree of uncolonized hard substrate (such as dead coral rubble). Identification of threshold values would be necessary. Farming of EA *E. denticulatum* only is recommended
Negative effects on natural seaweed habitats and indigenous eucheumatoid communities	Indirect	Avoid placing seaweed farms in the vicinity of natural seaweed areas. Identification of threshold values would be necessary. Farming of EA *E. denticulatum* only is recommended
Seaweed farms as vectors of pathogens/epiphytes, spread of associated introduced species	Indirect	Farming of EA eucheumatoids only. New introductions of foreign haplotypes should not be allowed

### Management recommendations concerning direct effects of seaweed farming

Since there are studies showing that seaweed farming can impact seagrasses negatively, it is recommended that not all seagrass meadows in an area are covered by seaweed farms. Also, different species of seagrasses tend to respond differently to seaweed farms (see Eklöf et al. 2006a), so that some seagrass meadows (consisting of *Thalassia hemprichii*) might be less vulnerable to farming activities than others (consisting of *Enhalus acoroides*). We would therefore recommend to place seaweed farms either on smaller, more resilient seagrass species, on sandy areas or potentially alternate between farming plots.

### Management recommendations concerning indirect effects of seaweed farming

To reduce indirect environmental effects by the spread of SEA haplotypes of eucheumatoids in the WIO area, it is recommended that more efforts should be made to identify native EA haplotypes of *E. denticulatum*, *K alvarezii* and other potential seaweed species, which are suitable/profitable for farming purposes. No more non-indigenous strains of either *E. denticulatum* or *K. alvarezii* should be allowed to be introduced for farming purposes before proper risk assessments and monitoring of the current situation have been made and farming management is safe for escapes and unintentional introductions. In that way, there is a limited risk of loss of genetic diversity among indigenous *E. denticulatum* populations.

In the absence of suitable native haplotypes for farming and since previous research shows that SEA *E. denticulatum/K. alvarezii* can have detrimental effects on reef-building corals of high structural complexity, it is recommended to not place farms of SEA haplotypes in close vicinity to reefs with branching *Acropora* spp. The exact distances away from corals might depend on hydrodynamic, biotic, and abiotic factors and should be designed accordingly.

Farms should further be avoided in areas where suitable settling substrate, in the form of coral rubble or rocky substrate with high rugosity, is available close to farms as these could provide ‘stepping stones’ for introductions of SEA seaweeds. It is recommended that farms are placed in areas which are dominated by soft substrate, preferable sand. Also, here, critical distances to hard substrate might be site-dependent and have to be evaluated further.

Because herbivores have the ability to induce top-down control on algal assemblages on coral reefs, it is recommended that seaweed farming should only be performed in areas where herbivorous fish communities are not depleted or overfished.

## Conclusions

The future of seaweed farming within the WIO area will likely depend on market value and market demand, and environmental impacts caused by farming will also likely be comparatively low; however, this is dependent on management choices. Consequently, environmental impacts by seaweed farming might be negligible, but certain issues need to be evaluated before this can be stated. Introduction of non-native seaweeds is a major concern. This is especially important for seaweeds like eucheumatoids, due to their ability to efficiently reproduce asexually by fragmentation, their fast growth rates, and the capacity to easily attach to substrate. In conclusion, any seaweeds—tropical or temperate—that possess those characteristics and are introduced in an environment with favorable conditions (such as temperature, salinity, presence of settling substrate, etc.) could potentially be at risk of spreading into its new habitat, why precautionary measures are always recommended. Therefore, there is a need to identify native species resources with farming potential and develop future farming systems based on these only, if open water cultivation systems are used. Farming of native seaweeds would decrease the risk of genetic loss of wild seaweed populations, although crop-to-wild gene flow is not excluded (unless several native haplotypes are farmed), for which spatial planning/placement of farms might be more eligible. Furthermore, the introduction and spread of non-native pathogens would be avoided if only EA haplotypes were farmed.

In addition, there is a need to establish better knowledge on the overall species interactions, where the specific prevailing traits, such as between corals and seaweeds during and after coral bleaching events, are of significant importance for local ecological resilience, especially in light of overall increasing environmental pressures (climate change, loss of biodiversity, pollution, etc.). The better the overall ecological understanding, the higher is the potential for implementing accurate strategies for improved seaweed faming and overall coastal management.

## References

[CR1] Almany GR (2004). Differential effects of habitat complexity, predators and competitors on abundance of juvenile and adult coral reef fishes. Oecologia.

[CR2] Anyango JO, Mlewa CM, Mwaluma J (2017). Abundance, diversity and trophic status of wild fish around seaweed farms in Kibuyuni, South Coast Kenya. International Journal of Fisheries and Aquatic Studies.

[CR3] Ask EI, Azanza RV (2002). Advances in cultivation technology of commercial eucheumatoid species: a review with suggestions for future research. Aquaculture.

[CR4] Ask, E.I., A. Batibasaga, J. Zertuche-González, and M. de San. 2003. Three decades of *Kappaphycus alvarezii* (Rhodophyta) introduction to non-endemic locations. In *Proceedings of the international seaweed symposium*, vol. 17, 49–57. Oxford: Oxford University Press.

[CR5] Bahr KD, Jokiel PL, Toonen RJ (2015). The unnatural history of Kane‘ohe Bay: Coral reef resilience in the face of centuries of anthropogenic impacts. PeerJ.

[CR6] Barrios J, Bolanos J, López R (2007). Blanqueiamento de arrecifes coralinos por la invasion de *Kappaphycus alvarezii* (Rhodophyta) en Isla Cubaga, Estada Nueva Esparta, Venezuela. Boletín del Instituto Oceanográfico de Venezuela.

[CR7] Bax N, Williamson A, Aguero M, Gonzalez E, Geeves W (2003). Marine invasive alien species: A threat to global biodiversity. Marine Policy.

[CR8] Bell J, Galzin R (1984). Influence of live coral cover on coral-reef fish communities. Marine Ecology Progress Series.

[CR9] Bergman KC, Svensson S, Öhman MC (2001). Influence of Algal Farming on Fish Assemblages. Marine Pollution Bulletin.

[CR10] Bindu MS, Levine IA (2011). The commercial red seaweed *Kappaphycus alvarezii*—an overview on farming and environment. Journal of Applied Phycology.

[CR11] Bixler HJ, Porse IA (2011). A decade of change in the seaweed hydrocolloids industry. Journal of Applied Phycology.

[CR12] Bonaldo RM, Hay ME (2014). Seaweed-Coral interactions: Variance in seaweed allelopathy, coral susceptibility, and potential effects on coral resilience. PLoS ONE.

[CR13] Bryceson I (2002). Coastal aquaculture developments in Tanzania: Sustainable and non-sustainable experiences. Western Indian Ocean Journal of Marine Science.

[CR14] Burkepile I, Hay ME (2006). Herbivore vs. nutrient control of marine primary producers: Context-dependent effects. Ecology.

[CR15] Buschmann AH, Camus C, Infante J, Neori A, Israel Á, Hernández-González MC, Pereda SV, Gomez-Pinchetti JL (2017). Seaweed production: Overview of the global state of exploitation, farming and emerging research activity. European Journal of Phycology.

[CR16] Cabrera R, Umanzor S, Díaz-Larrea J, Araújo PG (2019). *Kappaphycus alvarezii* (Rhodophyta): New record of an exotic species for the Caribbean Coast of Costa Rica. American Journal of Plant Sciences.

[CR17] Casas G, Scrosati R, Piriz ML (2004). The invasive kelp *Undaria pinnatifida* (Phaeophyceae, Laminariales) reduces native seaweed diversity in Nuevo Gulf (Patagonia, Argentina). Biological Invasions.

[CR18] Castelar B, de Siqueira MF, Sánchez-Tapia A, Reis RP (2015). Risk analysis using species distribution modeling to support public policies for the alien alga *Kappaphycus alvarezii* aquaculture in Brazil. Aquaculture.

[CR19] Castelar B, Reis RP, Moura AL, Kirk R (2009). Invasive potential of *Kappaphycus alvarezii* off the off the south coast of Rio de Janeiro state, Brazil: A contribution to environmentally secure cultivation in the tropics. Botanica Marina.

[CR20] Chandrasekaran S, Nagendran NA, Pandiaraja D, Krishnankutty N, Kamalakannan B (2008). Bioinvasion of *Kappaphycus alvarezii* on corals in the Gulf of Mannar. India. Current Science.

[CR21] Conklin EJ, Smith JE (2005). Abundance and spread of the invasive red algae, *Kappaphycus* spp., in Kane’ohe Bay, Hawai’i and an experimental assessment of management options. Biological Invasions.

[CR22] Conklin KY, Kurihara A, Sherwood AR (2009). A molecular method for identification of the morphologically plastic invasive algal genera *Eucheuma* and *Kappaphycus* (Rhodophyta, Gigartinales) in Hawaii. Journal of Applied Phycology.

[CR23] Cottier-Cook, E. J., N. Nagabhatla, Y. Badis, M. Campbell, T. Chopin, W. Dai, J. Fang, P. He et al. 2016. Safeguarding the future of the global seaweed aquaculture industry, 12 pp. United Nations University and Scottish Association for Marine Science Policy Brief. ISBN 978-92-808-6080-1.

[CR24] Courchamp F, Fournier A, Bellard C, Bertelsmeier C, Bonnaud E, Jeschke JM, Russell JC (2017). Invasion biology: Specific problems and possible solutions. Trends in Ecology & Evolution.

[CR25] Cowburn B, Samoilys MA, Obura D (2018). The current status of coral reefs and their vulnerability to climate change and multiple human stresses in the Comoros Archipelago, Western Indian Ocean. Marine Pollution Bulletin.

[CR26] Cullen-Unsworth LC, Nordlund LM, Paddock J, Baker S, McKenzie LJ, Unsworth RK (2014). Seagrass meadows globally as a coupled social–ecological system: Implications for human wellbeing. Marine Pollution Bulletin.

[CR27] Dahl M, Deyanova D, Lyimo LD, Näslund J, Samuelsson GS, Mtolera MSP, Björk M, Gullström M (2016). Effects of shading and simulated grazing on carbon sequestration in a tropical seagrass meadow. Journal of Ecology.

[CR28] Davidson AD, Campbell ML, Hewitt CL, Schaffelke B (2015). Assessing the impacts of nonindigenous marine macroalgae: An update of current knowledge. Botanica Marina.

[CR29] de la Torre-Castro M, Rönnbäck P (2004). Links between humans and seagrasses - an example from tropical East Africa. Ocean & Coastal Management.

[CR30] Dorenbosch M, Grol MGG, Christianen MJA, Nagelkerken I, van Dder Velde G (2005). Indo-Pacific seagrass beds and mangroves contribute to fish density and diversity on adjacent coral reefs. Marine Ecology Progress Series.

[CR31] Dy DT, Yap HT (2001). Surge ammonium uptake of the cultured seaweed, *Kappaphycus alvarezii* (Doty) Doty (Rhodophyta: Gigartinales). Journal of Experimental Marine Biology and Ecology.

[CR32] Eggertsen M, Chacin DH, Åkerlund C, Halling C, Berkström C (2019). Contrasting distribution and foraging patterns of herbivorous and detritivorous fishes across multiple habitats in a tropical seascape. Marine Biology.

[CR33] Eklöf JS, de la Torre Castro M, Adelsköld L, Jiddawi NS, Kautsky N (2005). Differences in macrofaunal and seagrass assemblages in seagrass beds with and without seaweed farms. Estuarine, Coastal and Shelf Science.

[CR34] Eklöf JS, de la Torre-Castro M, Nilsson C, Rönnbäck P (2006). How do seaweed farms influence local fishery catches in a seagrass-dominated setting in Chwaka Bay, Zanzibar?. Aquatic Living Resources.

[CR35] Eklöf JS, de la Torre-Castro M, Gullström M, Uku J, Muthiga N, Lyimo T, Bandeira SO (2008). Sea urchin overgrazing of seagrasses: A review of current knowledge on causes, consequences, and management. Estuarine, Coastal and Shelf Science.

[CR36] Eklöf JS, Henriksson R, Kautsky N (2006). Effects of tropical open-water seaweed farming on seagrass ecosystem structure and function. Marine Ecology Progress Series.

[CR37] Eriksson H, Wickel J, Alban J (2012). Coral bleaching and associated mortality at Mayotte, Western Indian Ocean. Western Indian Ocean Journal of Marine Science.

[CR38] FAO. 2018. The State of World Fisheries and Aquaculture 2018—Meeting the sustainable development goals. Rome. Licence: CC BY-NC-SA 3.0 IGO

[CR107] Ferreira CEL, Junqueira ADOR, Villac MC, Lopes RM (2009). Marine bioinvasions in the Brazilian coast: brief report on history of events, vectors, ecology, impacts and management of non-indigenous species. Biological invasions in marine ecosystems.

[CR39] Fröcklin S, Torre-Castro M, Lindström L, Jiddawi NS, Msuya FE (2012). Seaweed mariculture as a development project in Zanzibar, East Africa: A price too high to pay?. Aquaculture.

[CR40] Guillemin M-L, Faugeron DS, Destombe C, Viard F, Correa JA, Valero M (2008). Genetic variation in wild and cultivated population of the haploid-diploid red algae *Gracilaria chilensis*: How farming practices favour asexual reproduction and heterozygosity. Evolution.

[CR41] Gullström M, de la Torre Castro M, Bandeira SO, Björk M, Dahlberg M, Kautsky N, Rönnbäck P, Öhman MC (2002). Seagrass ecosystems in the Western Indian Ocean. Ambio.

[CR42] Halling C, Wikström SA, Lilliesköld-Sjöö G, Mörk E, Lundsør E, Zuccarello GC (2013). Introduction of Asian strains and low genetic variation in farmed seaweeds: Indications for new management practices. Journal of Applied Phycology.

[CR43] Hayashi L, Hurtado AQ, Msuya FE, Bleicher-Lhonneur G, Critchley AT, Seckbach J, Einav R, Israel A (2010). A review of Kappaphycus farming: Prospects and constraints. Seaweeds and their role in globally changing environments.

[CR44] Hayashi L, Bulboa C, Kradlofer P, Soriano G, Robledo D (2013). Cultivation of red seaweeds: A Latin American perspective. Journal of Applied Phycology.

[CR45] Hayashi L, Oliveira EC, Bleicher-Lhonneur G, Boulenguer P, Pereira RTL, von Seckendorff R, Shimoda VT, Leflamand A, Vallée P, Critchley AT (2007). Carrageenan analyses of *Kappaphycus alvarezii* (Rhodophyta, Solieriaceae) cultivated in different conditions in Ubatuba Bay, São Paulo, Brazil. Journal of Applied Phycology.

[CR46] Hedberg N, von Schreeb K, Charisiadou S, Jiddawi NS, Tedengren M, Nordlund LM (2018). Habitat preference for seaweed farming - A case study from Zanzibar, Tanzania. Ocean & Coastal Management.

[CR47] Hehre EJ, Meeuwig JJ (2015). Differential response of fish assemblages to coral reef-based seaweed farming. PLoS ONE.

[CR48] Hehre EJ, Meeuwig JJ (2016). A global analysis of the relationship between farmed seaweed production and herbivorous fish catch. PLoS ONE.

[CR49] Hill NAO, Rowcliffe JM, Koldewey HJ, Milner-Gulland EJ (2012). The interaction between seaweed farming as an alternative occupation and fisher numbers in the Central Philippines. Conservation Biology.

[CR50] Kamalakannan B, Jeevamani JJJ, Nagendran NA, Pandiaraja D, Krishnan Kutty N, Chandrasekaran S (2010). *Turbinaria* sp. as victims to *Kappaphycus alvarezii* in reefs of Gulf of Mannar, India. Coral Reefs.

[CR51] Kamalakannan B, Jeevamani JJJ, Nagendran NA, Pandiaraja D, Chandrasekaran S (2014). Impact of removal of invasive species *Kappaphycus alvarezii* from coral reef ecosystem in Gulf of Mannar, India. Current Science.

[CR52] Kuffner IB, Walters LJ, Becerro MA, Paul VJ, Ritson-Williams R, Beach KS (2006). Inhibition of coral recruitment by macroalgae and cyanobacteria. Marine Ecology Progress Series.

[CR53] Lirasan T, Twide P (1993). Farming *Eucheuma* in Zanzibar, Tanzania. Hydrobiologia.

[CR54] Luxton DM (1993). Aspects of the farming and processing of *Kappaphycu*s and *Eucheuma* in Indonesia. Hydrobiologia.

[CR55] Lyimo TJ, Mvungi EF, Lugomela C, Björk M (2006). Seagrass biomass and productivity in seaweed and non-seaweed farming areas in the East Coast of Zanzibar. Western Indian Ocean Journal of Marine Sciences.

[CR56] Mac Monagail M, Cornish L, Morrison L, Araújo R, Critchley AT (2017). Sustainable harvesting of wild seaweed resources. European Journal of Phycology.

[CR57] Mallea AJA, Villanueva FCA, Bernardi J, Cabrera R (2014). Ecological risk assessment of the introduction of exotic carrageenophytes in the tropical Western Atlantic. Journal of Applied Phycology.

[CR58] Mantri VA, Eswaran K, Shanmugam M, Ganesan M, Veeragurunathan V, Thiruppathi S, Reddy CRK, Seth A, A. (2017). An appraisal on commercial farming of *Kappaphycus alvarezii* in India: Success in diversification of livelihood and prospects. Journal of Applied Phycology.

[CR59] McClanahan TR (2017). Changes in coral sensitivity to thermal anomalies. Marine ecology progress series.

[CR60] McClanahan TR, Ateweberhan M, Graham NAJ, Wilson SK, Sebastian CR, Guillaume MM, Bruggemann JH (2007). Western Indian Ocean coral communities: Bleaching responses and susceptibility to extinction. Marine Ecology Progress Series.

[CR61] Merino G, Barange M, Blanchard JL, Harle J, Holmes R, Allen I, Allison EH, Badjeck MC (2012). Can marine fisheries and aquaculture meet fish demand from a growing human population in a changing climate?. Global Environmental Change.

[CR62] Mouritsen OG, Dawczynski C, Duelund L, Jahreis G, Vetter W, Schröder M (2013). On the human consumption of the red seaweed dulse (*Palmaria palmata* (L.) Weber & Mohr). Journal of Applied Phycology.

[CR63] Mshigeni KE (1984). The red algal genus Eucheuma (Gigartinales, Solieriaceae) in East Africa: An underexploited resource. Hydrobiologia.

[CR64] Msuya FE, Critchley AT, Ohno M, Largo DB (2006). The impact of seaweed farming on the social and economic structure of seaweed farming communities in Zanzibar, Tanzania. World seaweed resources: An authoritative reference system.

[CR65] Msuya FE, Buriyo A, Omar I, Pascal B, Narrain K, Ravina JJM, Mrabu E, Wakibia JG (2014). Cultivation and utilisation of red seaweeds in the Western Indian Ocean (WIO) Region. Journal of Applied Phycology.

[CR66] Muir, C.E., A. Sallema, O. Abdallah, D.D. Luca, T.R.B Davenport. 2003. *The dugong (Dugong dugon) in Tanzania: A national assessment of status, distribution and threat*, 31 pp. New York: Wildlife Conservation Society.

[CR67] Mwandya AW, Mtolera M, Pratap HB, Jiddawi NS, Richmond M, Francis J (2001). Macroalgae as biofilters of dissolved inorganic nutrients in an integrated mariculture tank system in Zanzibar. Marine science development in Tanzania and Eastern Africa.

[CR68] Nagelkerken I, van der Velde G (2002). Do non-estuarine mangroves harbour higher densities of juvenile fish than adjacent shallow-water and coral reef habitats in Curaçao (Netherlands Antilles)?. Marine Ecology Progress Series.

[CR69] Neilson B, Wall CB, Mancini FT, Gewecke CA (2018). Herbivore biocontrol and manual removal successfully reduce invasive macroalgae on coral reefs. PeerJ.

[CR70] Nordlund LM, Unsworth RK, Gullström M, Cullen-Unsworth LC (2018). Global significance of seagrass fishery activity. Fish and Fisheries.

[CR71] Obura, D., M. Gudka, F.A. Rabi, S.B. Gian, J. Bijoux, S. Freed, J. Maharavo, J. Mwaura et al. 2017. Coral Reef Status Report for the Western Indian Ocean (2017). In *Nairobi convention*. Global Coral Reef Monitoring Network (GCRMN)/International Coral Reef Initiative (ICRI): 144 pp.

[CR72] Ólafsson E, Johnstone RW, Ndaro SG (1995). Effects of intensive seaweed farming on the meiobenthos in a tropical lagoon. Journal of Experimental Marine Biology and Ecology.

[CR73] Phang S-M, Yeong H-Y, Lim P-E (2019). The seaweed resources of Malaysia. Botanica Marina.

[CR74] Poonian CNS, Lopez DD (2016). Small-Scale Mariculture: A potentially significant threat to dugongs (*Dugong dugon*) through incidental entanglement. Aquatic Mammals.

[CR75] Porse H, Rudolph B (2017). The seaweed hydrocolloid industry: 2016 updates, requirements, and outlook. Journal of Applied Phycology.

[CR76] Préat N, De Troch M, van Leeuwen S, Taelman SE, De Meester S, Allais F, Dewulf J (2018). Development of potential yield loss indicators to assess the effect of seaweed farming on fish landings. Algal Research.

[CR77] Renn D (1997). Biotechnology and the red seaweed polysaccharide industry: Status, needs and prospects. Trends in Biotechnology.

[CR78] Richards CL, Bossdorf O, Muth NZ, Gurevitch J, Pigliucci M (2006). Jack of all trades, master of some? On the role of phenotypic plasticity in plant invasions. Ecological Letters.

[CR79] Rodgers S, Cox EF (1999). Rate of spread of introduced Rhodophytes *Kappaphycus alvarezii*, *Kappaphycus striatum*, and *Gracilaria salicornia* and their current distribution in Kane’ohe Bay, O’ahu Hawai’i. Pacific Science.

[CR80] Rodrigueza MRC, Montaño MNE (2007). Bioremediation potential of three carrageenophytes cultivated in tanks with seawater from fish farms. Journal of Applied Phycology.

[CR81] Rönnbäck P, Bryceson I, Kautsky N (2002). Coastal aquaculture development in Eastern Africa and the Western Indian Ocean: Prospects and problems for food security and local economies. Ambio.

[CR82] Sagerman J, Enge S, Pavia H, Wikström SA (2014). Divergent ecological strategies determine different impacts on community production by two successful non-native seaweeds. Oecologia.

[CR83] Sangha, J.S., S. Kelloway, A.T. Critchley, B. Prithiviraj. 2014. Seaweeds (Macroalgae) and their extracts as contributors of plant productivity and quality. In *Advances in botanical research*, 189–219. Amsterdam: Elsevier. 10.1016/B978-0-12-408062-1.00007-X.

[CR84] Schaffelke B, Hewitt CL (2007). Impacts of introduced seaweeds. Botanica Marina.

[CR85] Sellers AJ, Saltonstall K, Davidson TM (2015). The introduced alga *Kappaphycus alvarezii* (Doty ex P.C. Silva, 1996) in abandoned cultivation sites in Bocas del Toro, Panama. BioInvasions Records.

[CR86] Shearer TL, Rasher DB, Snell TW, Hay ME (2012). Gene expression patterns of the coral *Acropora millepora* in response to contact with macroalgae. Coral Reefs.

[CR87] Sievanen L, Crawford B, Pollnac R, Lowe C (2005). Weeding through assumptions of livelihood approaches in ICM: Seaweed farming in the Philippines and Indonesia. Ocean & Coastal Management.

[CR88] Subasinghe R, Soto D, Jia J (2009). Global aquaculture and its role in sustainable development. Reviews in Aquaculture.

[CR89] Tano SA, Halling C, Lind E, Buriyo A, Wikström SA (2015). Extensive spread of farmed seaweeds causes a shift from native to non-native haplotypes in natural seaweed beds. Marine Biology.

[CR91] Tano SA, Eggertsen M, Wikström SA, Berkström C, Buriyo SA, Halling C (2016). Tropical seaweed beds are important habitats for mobile invertebrate epifauna. Estuarine, Coastal and Shelf Science.

[CR90] Tano SA, Eggertsen M, Wikström SA, Berkström C, Buriyo AS, Halling C (2017). Tropical seaweed beds as important habitats for juvenile fish. Marine and Freshwater Research.

[CR92] Taylor DI, Schiel DR (2010). Algal populations controlled by fish herbivory across a wave exposure gradient on southern temperate shores. Ecology.

[CR93] Thurber RV, Burkepile DE, Correa AM, Thurber AR, Shantz AA, Welsh R, Pritchard C, Rosales S (2012). Macroalgae decrease growth and alter microbial community structure of the reef-building coral *Porites astreoides*. PLoS ONE.

[CR94] Tiwari B-K, Troy DJ, Tiwari B-K, Troy DJ (2015). Seaweed sustainability—food and nonfood applications. Seaweed sustainability.

[CR95] Usandizaga S, Camus C, Kappes JL, Guillemin M-L, Buschmann AH (2018). Nutrients, but not genetic diversity, affect *Gracilaria chilensis* (Rhodophyta) farming productivity and physiological responses. Journal of Phycology.

[CR96] Usandizaga S, Camus C, Kappes JL, Guillemin M-L, Buschmann AH (2019). Effect of temperature variation in *Agarophyton chilensis*: Contrasting the response of natural and farmed populations. Journal of Applied Phycology.

[CR97] Valderrama, D. 2012. Social and economic dimensions of seaweed farming: A global review. In *IFET Tanzania proceedings*, 11 pp.

[CR98] Valderrama, D., J. Cai, N. Hishamunda, N.B. Ridler, and Food and Agriculture Organization of the United Nations (Eds.). 2013. Social and economic dimensions of carrageenan seaweed farming. FAO fisheries and aquaculture technical paper, 200 pp. Rome: Food & Agriculture Organization of the United Nations.

[CR99] Valero M, Guillemin M-L, Destombe C, Jacquemin B, Gachon C, Badis Y, Buschmann AH, Camus C (2017). Perspectives on domestication research for sustainable seaweed aquaculture. Perspectives in Phycology.

[CR100] Veiga P, Rubal M, Sousa-Pinto I (2014). Structural complexity of macroalgae influences epifaunal assemblages associated with native and invasive species. Marine Environmental Research.

[CR101] Vergés A, Alcoverro T, Ballesteros E (2009). Role of fish herbivory in structuring the vertical canopy algae *Cystoseira* spp. in the Mediterranean Sea. Marine Ecology Progress Series.

[CR102] Wijesinghe WAJP, Jeon YJ (2012). Biological activities and potential industrial applications of fucose rich sulfated polysaccharides and fucoidans isolated from brown seaweeds: A review. Carbohydrate Polymers.

[CR108] Xiao X, Agusti S, Lin F, Li K, Pan Y, Yu Y, Zheng Y, Wu J (2017). Nutrient removal from Chinese coastal waters by large-scale seaweed aquaculture. Scientific Reports.

[CR103] Yang Y, Liu Q, Chai Z, Tang Y (2015). Inhibition of marine coastal bloom-forming phytoplankton by commercially cultivated *Gracilaria lemaneiformis* (Rhodophyta). Journal of Applied Phycology.

[CR104] Zemke-White WL, Ohno M (1999). World seaweed utilisation: An end-of-century summary. Journal of Applied Phycology.

[CR105] Zohary D (2004). Unconscious selection and the evolution of domesticated plants. Economic Botany.

[CR106] Zuccarello GC, Critchley AT, Smith J, Sieber V, Lhonneur GB, West JA (2006). Systematics and genetic variation in commercial shape *Kappaphycus* and shape *Eucheuma* (Solieriaceae, Rhodophyta). Journal of Applied Phycology.

